# Performance of regional water purification plants during extreme weather events: three case studies from New South Wales, Australia

**DOI:** 10.1007/s11356-023-28101-y

**Published:** 2023-06-15

**Authors:** Adrian Hickey, Lalantha Senevirathna

**Affiliations:** 1 Public Works, Sydney, NSW Australia; 2 School of Computing, Mathematics and Engineering, Charles Sturt University, Bathurst, NSW 2795 Australia; 3 Gulbali Institute for Agriculture, Water and Environment, Charles Sturt University, Albury, NSW 2640 Australia

**Keywords:** Bushfire, Climate change, Climate extremes, Drought, Flooding, Water management, Water quality, Water supply

## Abstract

Climate change is altering weather patterns, which affects water supply systems globally. More frequent extreme weather events like floods, droughts, and heatwaves are impacting the availability of raw water sources that supply cities. These events can lead to less water, higher demand, and potential infrastructure damage. Water agencies and utilities must develop resilient and adaptable systems to withstand shocks and stresses. Case studies demonstrating the impact of extreme weather on water quality are important for developing resilient water supply systems. This paper documents the challenges faced by regional New South Wales (NSW) in managing water quality and supply during extreme weather events. Effective treatment processes, such as ozone treatment and adsorption, are used to maintain drinking water standards during extreme weather. Water-efficient alternatives are provided, and critical water networks are inspected to identify leaks and reduce system demand. Local government areas must collaborate and share resources to ensure that towns can cope with future extreme weather events. Systematic investigation is needed to understand system capacity and identify surplus resources to be shared when demand cannot be met. Pooling resources could benefit regional towns experiencing both floods and droughts. With expected population growth in the area, regional NSW councils will require a significant increase in water filtration infrastructure to handle increased system loading. Continuous research, regular strategy reviews, and innovative approaches are essential to ensure a secure and reliable water supply during future extreme weather events.

## Introduction

The profound impact of climate change is evident through substantial alterations in weather patterns, leading to far-reaching consequences for water supply on a global scale (Abbass et al. 2022; Islam & Karim 2019). The impact of these changes extends to the well-being, progress, and sustainability of communities, economies, and the environment (Kok et al. 2019; Organization 2020; Yin et al. 2021). Clean and safe water is a fundamental resource required for survival, and without it, communities cannot prosper, economies cannot thrive, and ecosystems cannot function properly.

Numerous researchers have consistently documented the escalating frequency of extreme weather events such as floods, droughts, and heatwaves in recent years (Clarke et al. 2022; Maxwell et al. 2019). These events directly impact the availability of raw water sources that supply water to urban areas. Floods can damage water treatment plants and distribution systems, while droughts reduce water availability, forcing water agencies to rely on expensive and energy-intensive alternative sources like groundwater. The impact of these events on water supply is significant and can result in issues such as reduced water availability, increased water demand, and potential infrastructure damage (Islam & Karim 2019; Tzanakakis et al. 2020). Droughts, for instance, can cause a significant decline in groundwater levels, leading to water shortages and competition amongst users. Therefore, it is essential to address the effects of climate change on urban water supply systems and agencies to ensure a stable and sustainable water supply for communities, economies, and the environment.

Moreover, extreme weather events can also affect water quality, which has far-reaching consequences (Khan et al. 2015; Michalak 2016; Zou et al. 2023). Higher temperatures and stormwater runoff can affect water quality, making the treatment of raw water more complex and expensive. Warmer temperatures can promote the growth of harmful microorganisms and increase the risk of waterborne diseases, while stormwater runoff can carry pollutants and contaminants into raw water sources, leading to contamination and increased treatment costs (Funari et al. 2012; Paerl & Huisman 2009; Raj Shivakoti et al. 2011; Senevirathna et al. 2022; Taebi & Droste 2004). Such changes can compromise the safety and reliability of water supplies, which, in turn, can lead to health risks and economic losses.

In response to extreme weather events and climate change impacts on urban water supply systems, water agencies and utilities need to develop strategies that create resilient and adaptive systems. These strategies consist of diverse water sources, improved water storage and distribution systems, increased water use efficiency and strengthened governance, planning, and community engagement (Head 2014; Lempert & Groves 2010; Organization 2009; Short et al. 2012). Water agencies are also designing water-efficient technologies like low-flow fixtures, drip irrigation systems, and water-efficient appliances while implementing water pricing policies that encourage efficient water use. Advanced and innovative water treatment technologies can also address variable water quality issues cost-effectively and efficiently while improving the overall resilience and sustainability of urban water supply systems. Additionally, water reuse and recycling programs can reduce the demand for freshwater resources, which can improve urban water security. Some water agencies and utilities are implementing strategies that include green infrastructure, like rain gardens, green roofs, and bioswales (Chaffin et al. 2016; Liao et al. 2017; Thomas & Houck 2020). Additionally, the adoption of water pricing policies that incentivise efficient water use can be helpful.

The lack of reported case studies highlighting the impact of extreme weather events on water quality and treatment presents an obstacle to developing resilient and adaptive water supply systems for specific geo-environmental conditions. This paper addresses this issue by documenting changes in water quality and the performance of water purification, as well as the challenges faced by water managers during three extreme weather events in New South Wales (NSW), Australia. NSW is situated on the east coast of the Australian mainland, bordered by Queensland to the north, Victoria to the south, South Australia to the west, and the Tasman Sea to the east, with a coastline stretching for over 2000 km. NSW encompasses a diverse range of landscapes, including arid regions, coastal areas, and high-altitude regions, making it particularly susceptible to extreme weather events such as prolonged droughts, heatwaves, bushfires, and floods (Hashim & Hashim 2016; Jegasothy et al. 2017). These events can have a significant impact on the quantity and quality of raw water sources, creating challenges for urban water supply systems in NSW. Many local government bodies in NSW do not directly manage the catchments for their dams. Instead, these services are provided by independent government bodies created by legislation to manage water resources and related services. Water treatment plants in NSW face numerous challenges in treating variable raw water qualities and ensuring a secure water supply during extreme events, including changes in raw water quality, increased demand during hot and dry periods, and infrastructure damage due to flooding or bushfires. To address these challenges, water supply agencies in NSW have implemented several strategies to manage the impact of extreme weather events on urban water supply systems.

The present study aims to investigate the impact of extreme climate conditions on urban water supply security by focusing on the experiences of municipal water purification plants in NSW, Australia. Specifically, the research question addressed in this paper is what lessons we can learn from recent extreme events in NSW about managing water resources and purification during extreme climate conditions. To achieve this goal, the paper presents three case studies that document the challenges faced by water purification plants during extreme weather events and highlight the importance of proactive planning, investment in resilient infrastructure, and adoption of adaptive management strategies to ensure safe and reliable drinking water availability. The paper provides valuable insights into the issues related to urban water supply security during extreme climate conditions by drawing on the experiences of water management authorities in NSW. By analysing the performance of water purification plants during extreme events, the paper identifies key challenges and opportunities for improving water management practices. The case studies presented in the paper demonstrate the importance of investing in resilient infrastructure to ensure continuity of service during extreme events. The paper also emphasises the need for proactive planning and adaptive management strategies that can help water management authorities to respond effectively to changing conditions. The insights presented in this paper can be useful for policymakers, water management authorities, and researchers interested in improving water management practices and ensuring safe and reliable drinking water availability during extreme events.

## Methodology

Data and information sourced to produce case studies conducted on the LGAs were collected through correspondence between council water resource managers and engineers. Raw and treated water quality parameters during the specified climate extreme were extracted from the council database to show the effectiveness of strategies and treatment methods. In addition, dam storage data were provided to determine supply and its effect on water quality. The data date ranges were 10-day periods selected to follow the climate extreme event’s timeline, in which water quality parameters would be affected.

## Case studies

The climate extremes of drought, bushfire, and flood were the focus of water management for this study, with three LGAs selected for case studies targeting one climate extreme for each council.

1. Drought-affected council (DAC)
2. Bushfire-affected council (BAC)
3. Flood-affected council (FAC)

The selection criteria used to determine the suitability of LGAs to be examined were as follows:

- The main town of the LGA must be a centre located within regional NSW.
- Climate extreme events of significance occurred within the last 5 years.
- Council was willing to support research and provide data and non-sensitive information.

Table 1 summarises the catchment and water quality characteristics of each study area.

**Table 1:** Catchment and water quality characteristics of each case study

	Drought-affected council (DAC)	Bushfire-affected council (BAC)	Flood-affected council (FAC)
Drinking water supply catchment characteristics	The DAC comprises six major creek catchments with a dedicated drinking water reservoir with a storage capacity of over 17,000 ML. It also gains additional inflows from a secondary water supply reservoir. The town’s water treatment plant (WTP) uses the conventional water treatment process, ozone treatment, and biological activated carbon (BAC) filtration to produce a potable supply for the township. The major catchments of the DAC are mainly used for agricultural purposes, including stock grazing and fruit-producing farmland.	The BAC catchment is situated amongst steep gradient sandstone and ironstone rock formations, covered with natural bushland. Water entering the dam occurs through sheet flow and filtering through rock, making soil addition through erosion generally minor. Water quality effects following a rain event generally occur within 3 days, with quality stabilisation occurring quickly due to the catchment’s nature and largely rock composition. Rainfall occurring months and years after a major bushfire continues to wash bushfire-affected material into waterways and causes ongoing water quality issues.	The FAC catchment area has both a high potential for salinity and erosion. It is estimated that 118,000 t of sediment would be washed into the catchment each year due to gully, sheet, and rill erosion. Willow trees have spread throughout the catchment after their introduction to stabilise riverbanks. Excessive willow debris resulting from high rain events has contributed to increased flood inundation and infrastructure damage. Due to the nature of a large catchment feeding the FAC’s town supply, the most change to water quality will occur within 3 days after a downpour. The FAC consists largely of an agricultural catchment contributing animal by-products, trees, and fertilisers to runoff entering the water supply system.
Water supply microbial issues	During the summer months of the recent droughts, the DAC reservoirs developed algal blooms due to rising water temperatures and higher nutrient levels. Reservoir circulators are positioned to address water stratification, aiming to prevent favourable conditions required for algae reproduction. Variable offtake trunnions enable water piped to the WTP to be sourced from advantageous depths, reducing the extent and need for treatment. To combat algal effects entering the WTP, such as taste and odour, the DAC uses ozone treatment to destroy pathogens present in the water.	Microbial activity affecting water quality is insignificant to the BAC catchments. They consist mainly of natural bushland and lack agricultural farmland, which contributes to faecal matter and excessive nutrients. Any microbial effects occurring within the BAC catchments are not considered related to bushfire events. The BAC monitors microbial levels within raw water and treats it with chlorination doses to control rare microbial occurrences. However, following bushfires, the BAC water managers use higher doses of chlorine to ensure water left stagnant in supply mains is not a source of microbial activity.	Of the microbial effects, *E. coli* has the greatest effect during flooding within the FAC catchment, largely due to stock defecating within watercourses giving the microorganism conditions suitable to thrive. In catchment areas, the FAC restricts access to stock through fencing. A destratification system which uses reservoir circulators prevents stratification and conditions suitable for microbial growth. In addition, the FAC pumps the water of the best quality out of the reservoir by having variable offtake trunnions to select water at the most advantageous depths.
Water supply physical and chemical issues	In drought, physical and chemical issues arise after rain transports matter built up in watersheds into storage. The DAC did not experience excessive effects in recent droughts, partly due to its location in a closed catchment with watersheds having an adequate ground cover that limited the amount of eroded material entering the system. The physical and chemical effects that occur are managed through conventional treatment.	Rains following bushfire events contribute greatly to both turbidity and colour change and changing pH levels for the raw water supply. Additional erosion can arise due to trees being uprooted and plant matter that binds soils being destroyed, exposing soils to concentrated runoff occurring on steep gradients. Due to limited access to the catchment, very little to no erosion and sediment strategies are used to reduce ash, soil, and other pollutants entering the system. The BAC used a larger quantity of chemical dosing during the Black Summer bushfires to account for the increased effects on water quality.	Turbidity and colour are two characteristics that are highly changeable within the FAC catchment. The FAC catchment soils are rich in manganese, which high rainfall events wash into the reservoir catchment and the rivers feeding the WTP. Manganese present in the water supply may result in a colour change to residents’ water treated before use. The FAC uses the conventional water treatment process, which involves three main stages—flocculation, filtration, and chlorination. The first two of these processes remove physical properties. A new filtration system was established to oxidise manganese further by using additional chemicals, including caustic soda and polymer, to initiate sedimentation.
Water supply infrastructure damage	Although the recent droughts did not cause infrastructure damage, the DAC invested in identifying and rectifying leaks within the water reticulation network and implementing additional water-saving systems on both residential and industrial levels.	Following the bushfire, debris mainly from trees, shrubs and other flora entered the water system. High rainfall events months and years after a major bushfire can continue to dislodge remnants, enabling larger debris to impact structures over a long period.	Over the years, flooding has caused damage through increased debris and erosion. The FAC implements flotation boom deflectors and trash racks to deflect debris during flooding from infrastructure such as the weir pool offtake at the WTP. Areas are patrolled to move unwanted accumulated debris and assess the need to increase erosion measures.

## Results

### Drought-affected council (DAC)

**Fig. 1:**
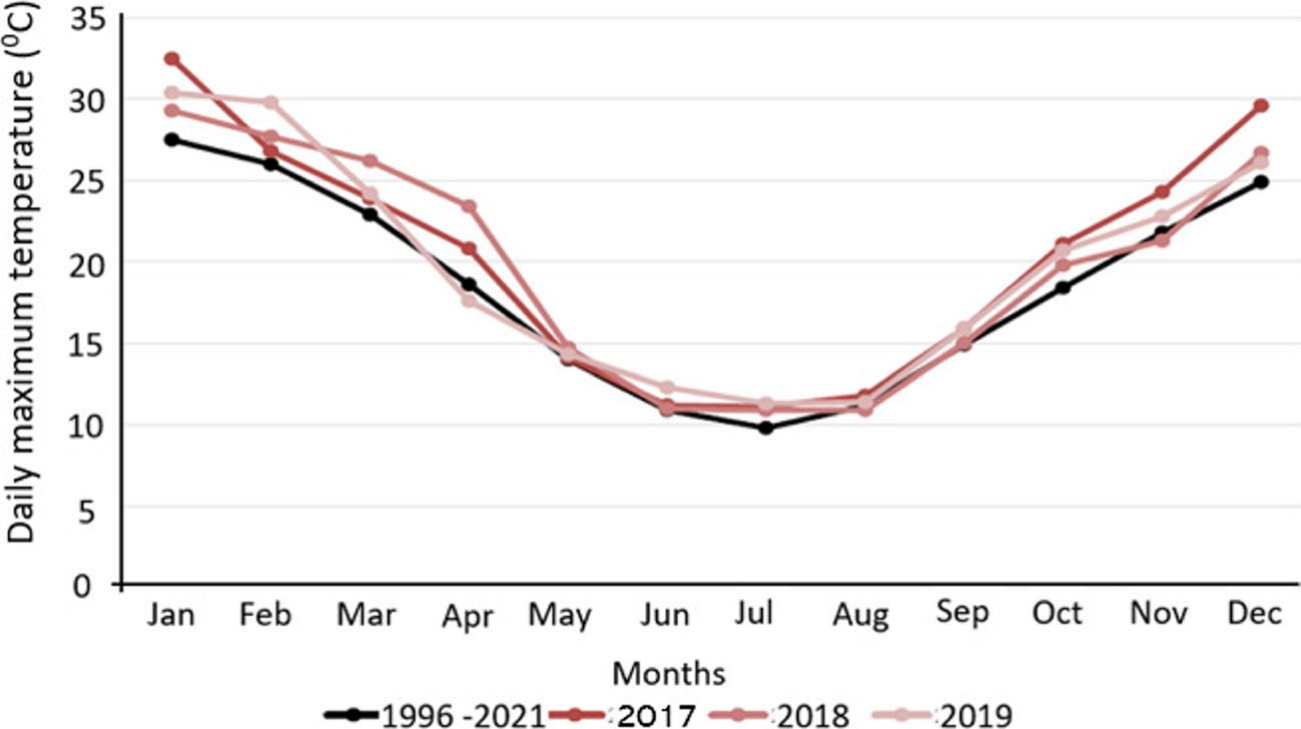
Monthly mean maximum temperatures recorded in 2017, 2018, and 2019 and historical average (1996–2021) 2017

During the 2017–2020 drought, the DAC airport weather station data showed that temperatures were well above average each year, with an annual average increase of 1.5 °C. In 2019, there was an annual increase of 1.9 °C, and the summer months increased by 3.5 °C, with January and December approaching 5 °C above average (Bureau of Meteorology, 2022). Due to evaporation and higher water consumption, the summer months featured the greatest loss in the water supply. Figure 1 demonstrates monthly temperature comparisons for the 2017–2020 drought and the average monthly temperatures since the DAC airport weather station records began (Bureau of Meteorology, 2022).

**Fig. 2:**
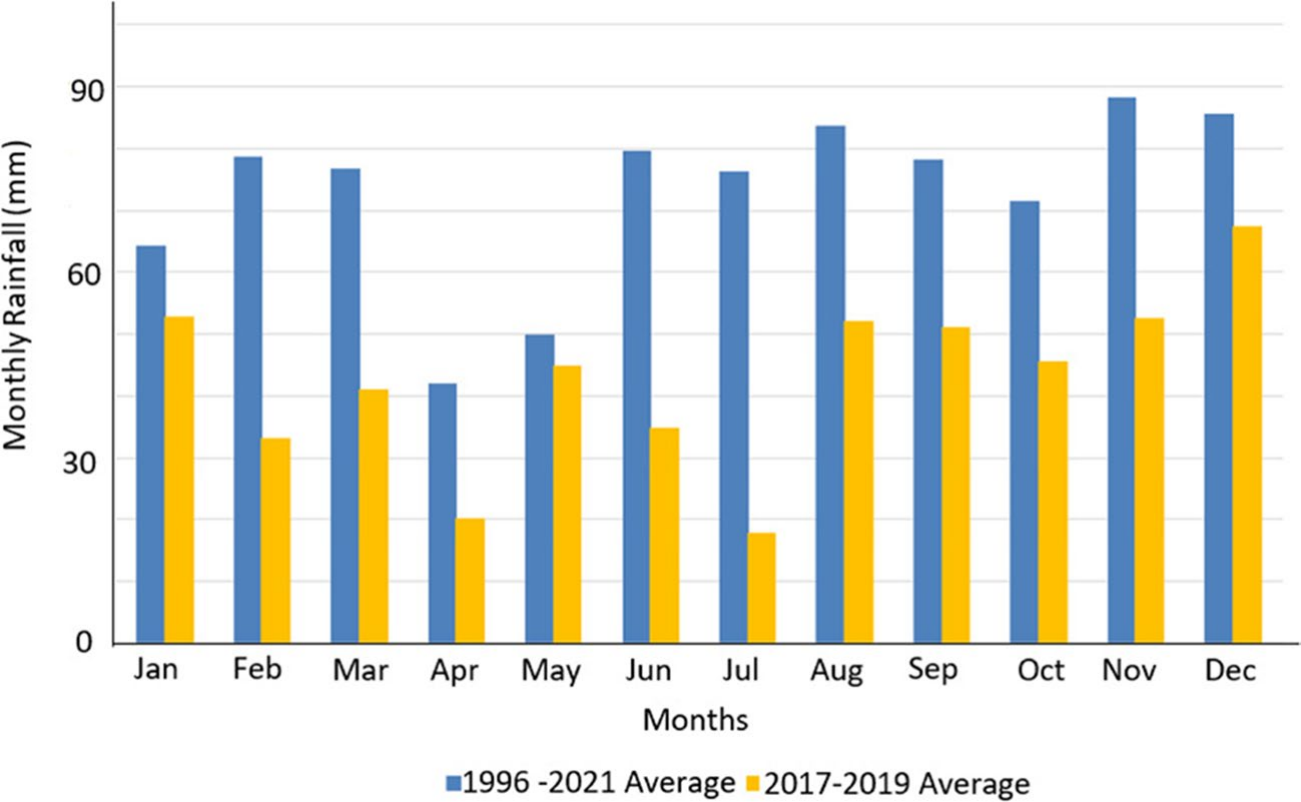
Monthly average rainfall recorded in 2017–2019 and 1996–2021, measured at DAC airport

According to DAC airport weather station data, during the 2017–2020 drought, the DAC averaged 512 mm of annual rainfall, with a low in 2019 of 425 mm. The DAC’s yearly average between 1996 and 2021 is 924 mm, equating to 412 mm above the recent 3-year drought period. Figure 2 shows monthly rainfall average comparisons for the 2017–2020 drought and the average monthly rainfall between 1996 and 2021 at the DAC airport weather station.

The DAC’s water supply design has resulted in several different water-saving and recycling schemes. In addition to existing dam supplies, the DAC has added additional sources to supplement the potable supply and reduce potable water use by operating other systems. The 2017–2020 drought saw the DAC’s residents reduce their average water consumption by 70 L/per day/per person. That equates to an average yearly saving per person of 25,550 L.

### Drought water quality and supply management

March 2016 was the beginning of a good year of rain where the DAC reservoir was above 60% capacity. During such times, there is significant dilution, reducing the concentrations of impurities compared to severe drought. The 10-day period in this month had two small rain events of between 10 and 15 mm and provided a basis for measuring water quality. September 2018, April 2019, and December 2019 were key milestones of the drought period as the DAC’s primary water source continually declined. The events were similar, which indicated potential declining water quality as the dilution of the serovar decreased. When the first rain was observed towards the end of the drought (75 mm of rain over 3 days), the reservoir storage levels were at their lowest. At this time, the DAC was operating under level 5 water restrictions as residents faced the potential “day zero” scenario of having the town’s water supply run dry. It was observed that a large portion of sheet flow was captured by farm dams, dry earth, and water networks upstream of the storage. However, flow entering the catchment brought eroded material, agricultural-related pollutants, and the general build-up of material left on the land during the drought when there was no rainfall cleansing events.

Without new or regular inflows to the main raw water reservoir, the DAC was confronted with water quality issues and algal production that occurred during the summer months, in addition to thermal stratification, causing temperature differentials and varying depths of water quality. To improve the raw water directed to the WTP, the DAC conducted vertical mixing with reservoir circulators and piped water from advantageous depths with variable offtake trunnions. This ensured that the raw water supplied and tested to the treatment plant was of higher quality and required less treatment. During the 2017–2020 drought, the DAC maintained all treated water to the ADWG standards. The DAC’s water treatment processes have ensured stability within water quality parameters to enable ADWG standards to be achieved. However, the supply caused concern for the DAC, with the primary potable water source reaching a low of 19.2% capacity in February 2020. The infrastructure built over the last 10 years and the implementation of a demand management strategy and water restrictions allowed the main township in the DAC to successfully manage its way through the millennium and 2017–2020 droughts.

### Drought lessons learned

During the 2017–2020 drought, the DAC encountered a scenario where the area was left with no water. This emphasised the effectiveness of the WTP in preserving water quality in pure storages and underlined the importance of considering alternative solutions to augment the township’s water supply.

DAC’s primary concern with respect to drought proofing is the limited water sources and storage capacity, which can be addressed through gaining access to alternative storage facilities outside the LGA or utilising other water sources within the LGA that are not designated as potable water sources, given that options within the LGA have already been extensively explored; to increase secure yield, further research is required, and constructing pipelines to larger dams external to the LGA and raising the dam wall of a nearby storage to increase capacity represent viable measures to enhance water security and bolster the town’s water supply during droughts, while DAC’s advanced treatment processes, particularly the ozone treatment and BAC filtration capabilities, have the potential to enable drinking water quality to be achieved in previously unviable sources, such as effluent, thereby optimising the DAC network and further increasing water security.

The DAC and its residents’ cooperative action to reduce water usage through audits, network improvements, and conscious water use ensured an adequate supply throughout the recent drought. Droughts will further educate the DAC as they have highlighted the effectiveness of the town’s water supply management. The benefits of the planning and implementation of the infrastructure proved capable of navigating the 2017–2020 drought; however, the reduction of the town’s storage capacity also displays the need to search for additional town water sources to provide further security.

### Bushfire-affected council (BAC)

**Fig. 3:**
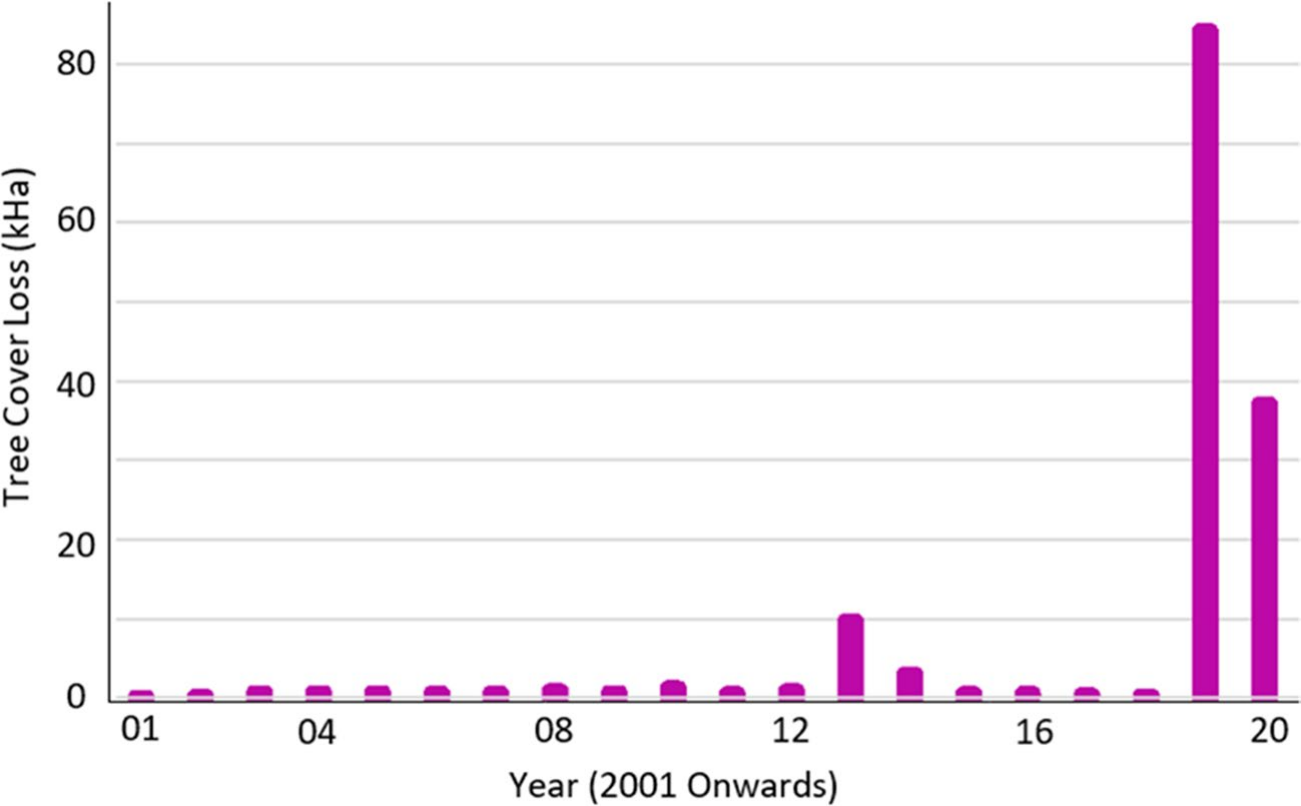
Magnitude of tree cover losses* in BAC due to bushfire events (2001–2020). *Tree cover is defined as all vegetation greater than 5 m in height and may take the form of natural forests or plantations across a range of canopy densities. Tree cover loss is defined as stand replacement disturbance, or the complete removal of tree cover canopy.

The BAC experiences an average of 128 bushfires and grassfires annually, with approximately three burning more than 20 ha and considered major fires. However, over 512,000 ha were burned in the bushfire in 2019–2020. The BAC’s Bushfire Management Risk Plan identifies Drinking Water Catchments as an economic asset at risk of future bushfires. The result of the bushfire was that 121,300 ha, or 46% of the council’s tree cover, was lost in addition to grassed and open areas. Figure 3 displays the extent of tree cover lost within the BAC due to fires between 2001 and 2020.

Following the driest year on record (2019), with 466.2 mm falling across the year, there were minor rain events in January, followed by drought-breaking rains throughout February, which helped extinguish bushfires across the region. August 2019 was at the end of the drought; however, the BAC’s reservoir was still above 80% capacity. In this period, there was significant dilution, reducing the concentration of impurities compared to lower dam levels. The 10-day period has very little rain and provides a basis for water quality where water is not mixed. January 2020 had 40.6 mm of rain falling across 5 days during the bushfire. At this point, the BAC’s reservoir has fallen to below 35%. This event allowed a review of a burning bushfire’s effect on water quality. A bushfire extinguishing high rainfall event occurred in February 2020, when 111.0 mm of rain fell across 4 days, including a high of 51 mm in a day. The heavy rains transported burnt matter and debris into the storage and increased the dam’s storage from 45.3 to 100%. Another rainfall event 3 weeks after the bushfire, in March 2020, produced 35.8 mm falling over 3 days. The heavy rains continued transporting bushfire-affected matter into the water storage system. In January 2022, following another bushfire, 153.5 mm of rain fell across 9 days, including 75.0 mm in 1 day.

### Bushfire water quality and supply management

The results show that during the 2019–2020 Black Summer bushfires, the BAC maintained all treated water to the ADWG standards. The BAC’s water treatment processes have ensured stability within water quality parameters to achieve ADWG standards. The data shows the BAC’s effectiveness in managing water quality throughout a bushfire—the treatment processes and raw water were at manageable levels and effectively treated for each parameter. It is noted that the BAC raw water was typically acidic, reaching pH levels as low as 5.56. For the treated water, the pH ranged between 6.95 and 8.40 within the tested ranges. The apparent colour is an important aesthetic characteristic for customer acceptance. For raw water, the apparent colour was typically not below 100 HU (Hazen units). Aside from 469 HU following the extinguishing rains in February 2020, the worst period occurred more recently in January 2022, when HU values within the 10-day period were all above 260. The colour ranged between 0 and 10 HU within the tested ranges for the treated water. This range meets the requirements set within the ADWG. Tested raw water turbidity levels generally did not see a direct correlation with heavy rainfall. The highest reading for the data shown was 41.90 NTU in February 2020. The BAC treatment processes successfully maintained the treated water within the ADWG throughout the identified period. However, these readings have extended above 0.5 NTU, with a maximum reading of 0.85 NTU, nearing the ADWG standard of less than 1 NTU. The supply within the BAC reservoir dropped significantly in the lead-up and during the bushfire. In August 2019, the reservoir was at over 80.0% capacity. Five months later, in January 2020, the capacity was less than 35%. The rains that extinguished the bushfire just 2 weeks later saturated the catchment and replenished the storage to 100%. This shows the catchment’s ability to recharge following significant rainfall.

The BAC’s key challenges for water management following a bushfire are catchment management and pre-WTP treatment or filtration. The BAC reservoir and surrounding catchment area are within steeply sloped rock formations and dense bushland. This creates access issues and is unfavourable to the initial and continual response to a bushfire where treatments cannot be implemented, and debris is removed from the site. As the BAC does not undertake pre-WTP water treatment or filtration, the raw water entering the WTP is unregulated. Therefore, the raw water requires additional chemical dosing and treatment to meet ADWG standards. Although the BAC maintained supply throughout the period, the extent of additional supply changed quickly with residents’ early preparation to wet down areas to green landscaped areas and pre-empt ember attacks. The event proved that the supply change timeframe occurs over far longer periods than previously expected by the BAC water managers, as residents act and prepare following events elsewhere in the country. The historic event will enable a greater understanding and reference for regional water managers to future events. The BAC has concerns about future bushfire events and associated water management issues due to the inaccessible nature of the catchment. The dense, steep terrain will make it difficult for operators, rangers, and water managers to maintain and manage events following a bushfire. The lack of accessibility also negates the ability to implement pre-storage water quality treatments and catchment recovery and monitoring procedures.

The BAC has an opportunity to adopt pre-storage water quality treatments to improve water quality before pumping to the WTP. Additionally, there is no treatment or filtration between the storage and WTP other than drawing water from advantageous depths with variable offtake trunnions. Therefore, an overall opportunity to implement strategies to improve raw water quality is available. This would improve the health of the catchment and the BAC’s storage dams and reduce the extent of chemical dosing required to meet the ADWG standards. Increased accessibility to the site and its surroundings would improve catchment management following a bushfire and benefit the monitoring and maintenance of the water supply infrastructure.

### Floodwater-affected council (FAC)

**Fig. 4:**
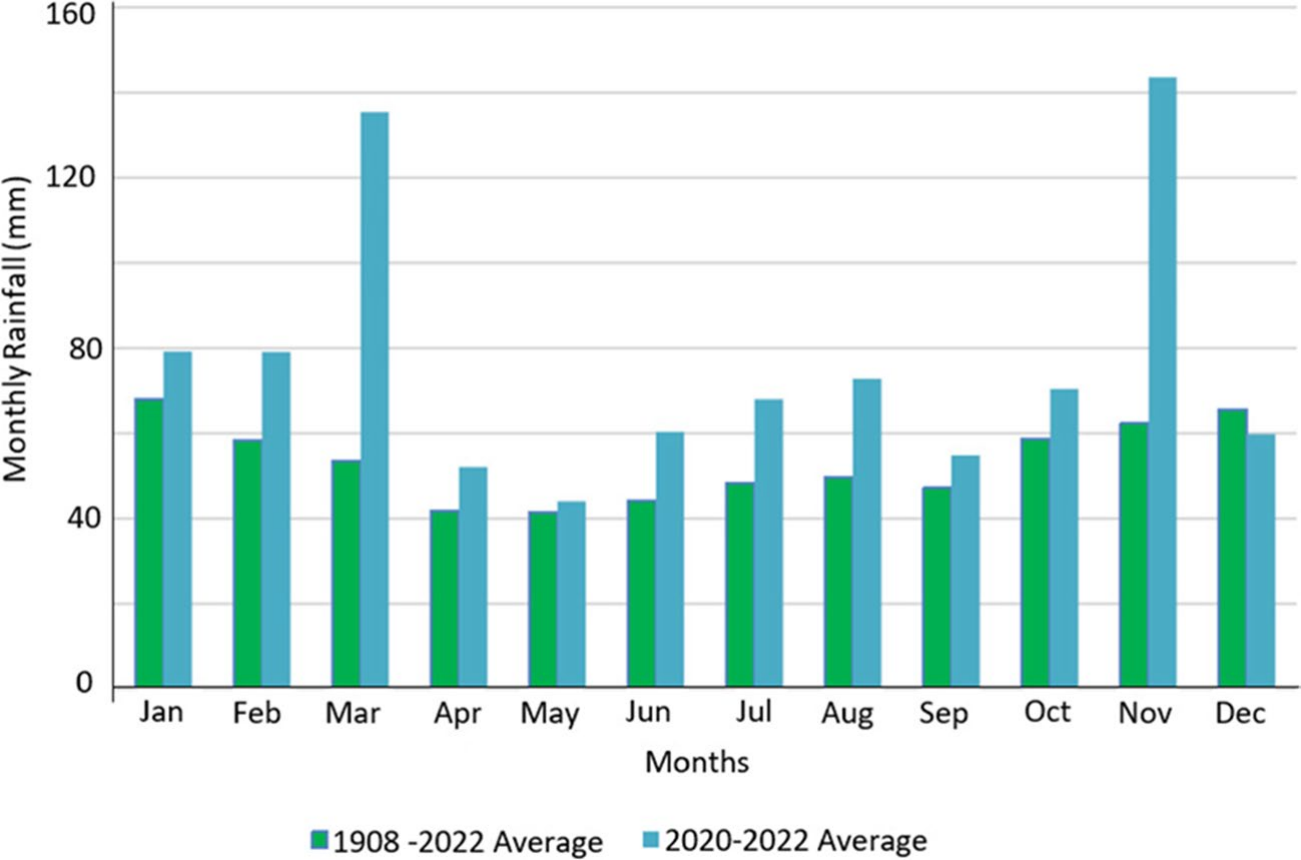
FAC rainfall between 2020 and 2022 vs. historic averages

In 2021, the FAC endured its third wettest year since records began in 1908, with 977.2 mm compared to FAC’s annual average rainfall of 639.7 mm. In addition, November 2021 was the wettest month in the FAC’s history, with 142.4 mm, far greater than the November average of 65.6 mm. Figure 4 shows monthly rainfall comparisons for 2020–2022 and the average monthly rainfall between 1908 and 2022 at the FAC Weather Station. Except for December, between 2020 and 2022, all monthly rainfalls exceeded the long-term average monthly rainfalls, with both March and November recording more than double the long-term averages.

### Flood water quality and supply management

In March 2020, the FAC recorded its first rainfall event greater than 60 mm in 14 months. This followed three consecutive years where only 75% of the average annual rainfall was reached. At this stage, the FAC’s reservoir was at about 30% capacity. High rainfall events were reported in March 2021 (135 mm) and January 2022 (80 mm), when reservoir capacity exceeded 100%. November 2021 was the wettest month in the FAC’s history. The FAC’s catchment was saturated before the rainfall occurred, and the reservoir exceeded full capacity. To provide context for the 10-day data period for water quality following rainfall events, Table 2 shows the average daily raw and treated water quality and ADWG maximum values for the selected water quality parameters.

**Table 2:** Maximum and minimum values for raw and treated parameters

	Raw water				Treated water		
	*E. coli*	pH	Colour	Turbidity	pH	Colour	Turbidity
	CFUs/100 mL	pH	HU	NTU	pH	HU	NTU
Max	>16,000	8.01	553	298.00	8.37	5.00	0.25
Min	26.0	6.4	37.0	6.9	6.8	0.0	0.0

The FAC did not supply *E. coli* data and instead stated *E. coli* is not measured in the final water and is assumedly zero due to treatment (chlorine). This is verified through our reticulation monitoring

From the floods following the drought-breaking rains in 2020 to the present day, the FAC has maintained all treated water to the ADWG standards. Following the treatment processes, the data shows the FAC’s effectiveness in managing water quality throughout high rainfall. The *E. coli* during flooding often rose significantly above the normal range expected. For example, *E. coli* level was reported as >16,000 CFUs/100 mL in a day in November 2021. However, the following day, the value was 790 CFUs/100 mL. For the treated water, the pH ranged from 6.8–8.37. This range approaches the outer bounds accepted by the ADWG standards making it acceptable.

Turbidity and colour are two characteristics that can vary greatly within the FAC catchment area. This variability is caused by existing sediments being stirred up in watersheds and rivers, new materials washing into waterways, and an increase in erosion. The soils in FAC catchments are rich in manganese, which can be washed into the storage dam catchment and rivers feeding the WTP during high rainfall events. Manganese in the water supply can result in a change in colour for residents’ water once it is treated. To address this issue, a new filtration system was established in 2014 that utilises additional chemicals, such as caustic soda and polymer, to initiate sedimentation and further oxidise manganese. After sedimentation, additional dosing with caustic soda and sodium hypochlorite causes manganese to change from soluble to insoluble, and filters are then able to remove the remaining manganese. It is important to note that during flooding, FAC has adequate potable storage to allow the WTP to cease treatment during periods when water quality is at its worst. This has allowed for less processing and treatment, enabling raw water to stabilise before the WTP continues operation. The average colour of the raw water was 68 HU. Following flooding events in data captured, colour typically increased, with a high Hazen unit of 553 in November 2021. At the water treatment plant, the treated water was found to exhibit a colour range falling between 0 and 5 HU within the tested ranges. This range meets the requirements set within the ADWG. Raw water turbidity levels generally showed a significant spike immediately following a high rainfall event. The highest reading for the data shown was 298.00 NTU in March 2021.

The recent historic flooding events have proven that the dam’s storage capacity is sufficient to stop major flooding, as the systems in place can release water in a controlled manner to reduce the risk to downstream properties. In addition, the flooding has proven that there is currently sufficient potable storage to enable the WTP to cease treatment during flooding periods where water quality is at its worst. This has enabled less processing and treatment, allowing raw water to stabilise before the WTP continues operation. With extensive population growth projected in the area, the FAC will need a significant increase in water filtration infrastructure to manage the increased system loading. Currently, the network is serviced by one WTP, and residential areas are spreading, likely requiring further infrastructure to accommodate the growth. The current system is remarkable in that it boasts sufficient potable storage capacity to necessitate reduced processing and treatment. As a result, the raw water can stabilise before the WTP resumes its operation.

A series of levee banks in the FAC are designed for a 1/1000-year flood event. Climate change and the increase of record-breaking events leave uncertainty in the operation of flood mitigation infrastructure for councils across the country, including the FAC. Over time, managing the treatment process of raw water has required further advanced technology and stringent treatment processes to keep up with the ADWG and reduce raw water quality. The mark left on raw water quality by human actions has continued to reduce the quality of water entering the WTP. The FAC is on a flood plain, with large areas of floodwaters inundating the landscape in periods of high rainfall. Water harvesting could be implemented during flooding to redirect floodwaters to storage reservoirs or catchments to boost storage in dry spells. In addition, looking at alternative storage could provide a location to capture water harvested during floods, which would require further studies.

## Discussion

In NSW, 81% of the land is agricultural farmland, much of which overlaps water catchments, particularly in regional NSW (Department of Agriculture, 2021). The runoff from agriculture greatly pollutes water following flooding, as high concentrations of pollutants build up on farms over long periods. Animal by-products, fertilisers, and pesticides are examples of pollutants attributed to farmland (Xia et al. 2020). The effects of these substances entering waterways include increased microorganism production and distortion in the water’s chemical and physical properties (Howe & White 2003). The microbial effects of flooding are evident, as is the case for *Escherichia coli* (*E. coli*), where studies have shown that the highest values are driven by surface runoff. This is mainly due to faecal matter on watersheds being washed into waterways feeding town water supplies (Clarke et al. 2017). During flooding events, suspended sediment concentration in water is far greater than in times of drought or low flow. This occurs due to heightened soil erosion with water washing soil from cultivated lands, uncultivated pastoral lands, and channels and gullies (Wallbrink et al. 1998). The effect of rainfall on water quality and turbidity depends on various factors, including the state and size of the catchment and the intensity of the rainfall event (Fitzgerald et al. 2014). Sediment significantly raises turbidity levels and occurs at a higher rate when intense storms or rainfall events occur (Sun et al. 2001). In addition to the finer pollutants and particles that floods carry into rivers and water bodies, debris is carried into systems during flooding events. Debris can damage and create blockages to infrastructure, causing water management issues. A flood’s intensity contributes to the velocity of water within rivers and its capability to carry debris (Kalendher et al. 2016).

In addition to the benefits of replenishing catchments that provide long-awaited recharge to rivers and reservoirs, the floods caused significant damage to infrastructure and eroded soil left vulnerable by the drought and bushfires. Droughts leave behind residual effects, which become prevalent if followed by flooding. The water quality is affected significantly as concentrated sheet flow provides a mode of transport for eroded material and chemical and physical pollutants (Delpla et al. 2009). Increased precipitation in combination with baron landscapes meant the higher runoff rates provided little resistance to floodwaters as water sheeted across exposed catchments. As a result, heavy rainfall often compounds the effects of extreme weather events before impacting water quality to a greater extent (Fitzgerald et al. 2014). A flood following a drought makes water bodies susceptible to blackwater events as years of organic matter in leaf litter build up. Blackwater events occur through floods washing the organic material into water bodies and allowing carbon to egress into the water, elevating dissolved organic carbon levels. The increased levels of carbon allow a means for increased microorganism growth, which uses up dissolved oxygen within the water body. Although blackwater events are part of natural ecology, the severity of events is increased due to a changing climate. Following extended periods of drought, floods make blackwater events detrimental to water quality and leave water bodies susceptible to further events such as fish kills (Howitt et al. 2007). Rainfall subsequently transports an abundance of matter into waterways and storage, reducing raw and final water quality (Fitzgerald et al. 2014).

Regional LGAs’ preparation and response to drought are essential to resilience following periods of drought. Improving an organisation’s technical knowledge, in-house expertise, and capability and learning from past events on local, national, and international levels can benefit outcomes following drought (Wright et al. 2014). Monitoring water quality levels within a system and implementing strategies to address common water quality themes associated with drought, including turbidity, taste and odour, higher chemical and physical concentrations, and pathogen concerns, will greatly assist in maintaining compliance with standard regulations. In drought, trace element concentrations are greater due to a lack of inflow and decreased dilution. Studies have shown that water stratification and settlement cause varying depths to contain higher levels of some water quality characteristics, such as trace elements. Supply intake levels that can operate at varying depths enable the supply of raw water with better quality. Exploring improved intake structures may be beneficial to block or use shallow/deeper water depending on the water quality at various depths (Wright et al. 2014). Vertical mixing has proven to be an important control of the growth of microorganisms such as cyanobacteria or blue-green algae for several decades. The circulators disturb the conditions favourable to eutrophication. Artificial mixing disrupts stratification in reservoirs in drought by creating vertical diffusion (Han et al. 2020). As cyanobacteria thrive in slow-moving warm, nutrient water and can remain buoyant to use sunlight to create a food source, mixing the water disrupts these conditions (Funari et al. 2012).

Organic matter is a forerunner to increased levels of disinfection by-products (DBP) and total organic carbon (TOC), which are increased following rainfall proceeding a drought (Yang et al. 2008). Raised DBP and TOC occur due to increased organic matter washed into the network interacting with the disinfectants used in the water treatment. Spikes in organic matter can be avoided with vegetation control on reservoir embankments, limiting the amount of organic matter built up over extended periods of drought (Wright et al. 2014). To manage water quality during drought, water quality monitoring is essential to enable quick adjustments (Wright et al. 2014) to comply with regulatory standards. Monitoring and documenting for historical and reflective purposes provide lessons learned and opportunities for improvements during future events.

## Conclusions

Effective treatment processes, including ozone treatment and adsorption, alongside conventional methods, have maintained drinking water quality standards during extreme weather events. Thorough audits of vital water distribution networks identify leaks and offer water-efficient alternatives to reduce system demand. By implementing sufficient infrastructure, demand management strategies, and water restrictions, the township successfully navigated through the challenging weather conditions of the millennium and 2017–2020. To enhance drought resilience across NSW regions, collaboration and resource-sharing amongst local government areas, particularly through organisations like CENTROC, should be prioritised.

A systematic study is necessary to understand the capacity of water systems, considering each LGA’s storage capacity, system demand, and ability to withstand recent drought conditions. This study can help identify surplus resources that can be shared across regional NSW where demand cannot be met. Research on regional NSW consumers’ perceptions of treated effluent as a potential drinking water source would provide valuable insights. Additional case studies are needed to provide a more comprehensive understanding of drought-affected areas in regional NSW, taking into account different catchment characteristics and agricultural practices.

The water quality effects of bushfires were primarily physical and chemical, with minimal microbial impacts. The water treatment processes employed by the BAC involved increased chemical dosing to stabilise turbidity, colour, and pH levels after the bushfires, in addition to conventional treatment methods. Post-bushfire results showed higher turbidity and colour compared to pre-bushfire levels. Considering the likelihood of future extreme bushfire events, it is crucial to improve dam and catchment accessibility in affected areas and implement pre-treatment strategies such as land and channel treatments.

The case studies reviewed highlight how regional NSW councils have effectively handled water quality and supply in the face of diverse climate extremes over the past 5 years. However, with the evolving regional landscape and escalating climate volatility, it is crucial to prioritise ongoing research, continuous review, innovative strategies, and collaboration at both regional and state levels. These measures are vital to safeguard the well-being of residents in regional NSW. To ensure a secure and uninterrupted water supply during extreme weather events, it is imperative for central and state governments, as well as authorities, to allocate increased funding support for activities like research, case studies, and the development and adaptation of innovative technologies.

### Recommendations

Due to the volatile nature of regional NSW and the occurrence and resounding effects of drought, there is an opportunity to water harvest from floods to further droughtproof the region. To redirect floodwaters, local councils could use a series of levee banks located around town as a capture point to pump to storage. Further studies should be undertaken into securing this resource to supplement storage, particularly when storage levels are low, or whether another storage could be established to serve this event. With extensive population growth projected in the area, regional NSW councils will need a significant increase in water filtration infrastructure to manage the increased system loading. Currently, most regional councils depend on single water treatment facilities, and the reach of residential areas is spreading, which will likely require further infrastructure to accommodate the growth. As many regional towns may suffer both flood and drought over extended periods, pooling resources could benefit the LGA of concern and neighbouring LGAs, where a town may not have the resources to manage an event. Different locations using alternate strategies and treatments would provide insight into what has been done successfully elsewhere and other councils’ issues. Council collaboration and further research into water management during extreme weather events are key to future practices.

## Data Availability

Not applicable.
